# Transcriptional and Epigenetic Regulatory Mechanisms Affecting HTLV-1 Provirus

**DOI:** 10.3390/v8060171

**Published:** 2016-06-16

**Authors:** Paola Miyazato, Misaki Matsuo, Hiroo Katsuya, Yorifumi Satou

**Affiliations:** International Research Center for Medical Sciences, Center for AIDS Research, Priority Organization for Innovation and Excellence, Kumamoto University, Kumamoto 860-0811, Japan; pmiyazat@kumamoto-u.ac.jp (P.M.); 163r5152@st.kumamoto-u.ac.jp (M.M.); hkatsuya@kumamoto-u.ac.jp (H.K.)

**Keywords:** HTLV-1 2, provirus 3, retroviral latency

## Abstract

Human T-cell leukemia virus type 1 (HTLV-1) is a retrovirus associated with human diseases, such as adult T-cell leukemia (ATL) and HTLV-1-associated myelopathy/Tropic spastic paraparesis (HAM/TSP). As a retrovirus, its life cycle includes a step where HTLV-1 is integrated into the host genomic DNA and forms proviral DNA. In the chronic phase of the infection, HTLV‑1 is known to proliferate as a provirus via the mitotic division of the infected host cells. There are generally tens of thousands of infected clones within an infected individual. They exist not only in peripheral blood, but also in various lymphoid organs. Viral proteins encoded in HTLV-1 genome play a role in the proliferation and survival of the infected cells. As is the case with other chronic viral infections, HTLV-1 gene expression induces the activation of the host immunity against the virus. Thus, the transcription from HTLV-1 provirus needs to be controlled in order to evade the host immune surveillance. There should be a dynamic and complex regulation *in vivo*, where an equilibrium between viral antigen expression and host immune surveillance is achieved. The mechanisms regulating viral gene expression from the provirus are a key to understanding the persistent/latent infection with HTLV-1 and its pathogenesis. In this article, we would like to review our current understanding on this topic.

## 1. Introduction

It has been estimated that Human T-cell leukemia virus type 1 (HTLV-1) has been infecting humans for several thousand years [1]. In ancient times, prior to the advent of blood transfusions or drug abuse, the virus spread either by vertical transmission, from mother to child via breast-feeding, or by horizontal transmission mainly from man to woman, through sexual intercourse. HTLV-1 generally induces *de novo* infection not via free viral particles but via cell-to-cell contact between infected and uninfected cells [2,3,4]. The presence of infected lymphocytes in breast milk or sperm is pivotal for *de novo* infection. In the case of vertical transmission, infected individuals acquire the virus during infancy and need to carry the virus for decades, before they are able to transfer the virus to their children. Also, infected women need to remain healthy for decades in order to become pregnant, in spite of the persistent HTLV-1 infection in their bodies. To achieve such a long-term persistent infection without having severe health problems, HTLV-1 seems to have developed a strategy to achieve an asymptomatic condition by minimizing the effect of viral infection on our vital systems. At the same time, as with other viruses that cause persistent infections, HTLV-1 needs to evade the host immune surveillance [5].

**Figure 1:**
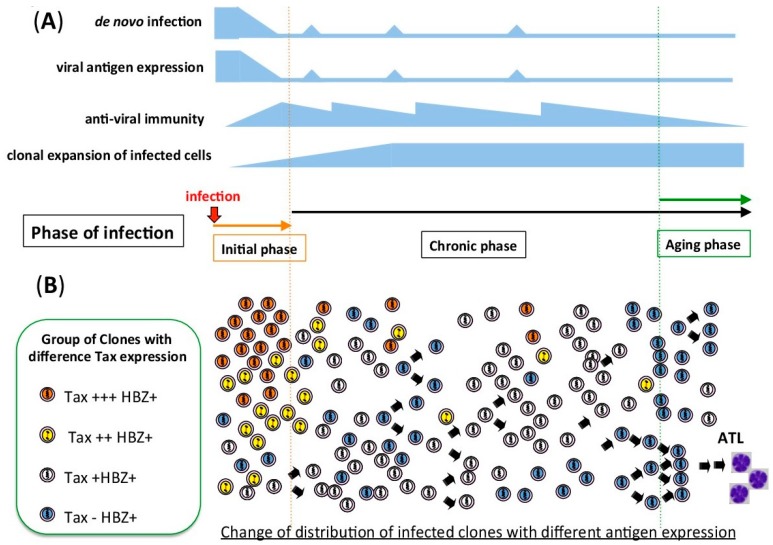
Schematic figure of Human T-cell leukemia virus type 1 (HTLV-1) infection from the initial to the chronic phase of infection. (**A**) During the initial phase of infection, before an anti-virus immunity has been established, *de novo* infection should be more dominant than the clonal expansion of infected cells. In the chronic phase of infection, antiviral immunity removes infected cells with high viral antigen expression. HTLV-1 increases the viral copy number by clonal expansion of the infected cells. There is sporadic viral antigen expression, which should maintain the activity of the anti-viral immunity. (**B**) Change in the distribution of infected clones with different antigen expression. In the initial phase, the proportion of infected clones with high Tax expression is high, because there is little anti-viral immunity. After the establishment of an anti-viral immunity, clones with high antigen expression are eliminated by the host immune system.

The main routes of *de novo* HTLV-1 infection are cell-to-cell transmission and/or extracellular biofilm-like-structure-mediated transmission [2,4]. In either case, the virus produces viral proteins that are necessary for reverse transcription and integration of the viral DNA into the host cellular DNA. HTLV-1 is required to keep a latent state in the chronic phase of infection, but it also needs to reactivate viral gene expression for *de novo* infection. This implies that the virus makes use of a reversible system to switch from the latent phase of infection to the active phase, where viruses are produced. For example, when the infected cells are transferred from mother to child, proviruses in the infected lymphocytes contained in the breast-milk would be transcriptionally reactivated, and producing infectious viral particles or inducing cell-to-cell transmission. Since an anti-virus immunity has not been established yet in the new host during the initial phase of infection, the virus would be able to spread via *de novo* infection (Figure 1).

In a typical asymptomatic carrier, approximately 2% of peripheral mononuclear cells are infected with HTLV-1 [6], which is far more frequent when compared with the proviral load of HIV‑1 in patients undergoing anti-retroviral therapy [7]. We can also observe cytotoxic T-lymphocytes (CTLs) specific for HTLV-1 antigens in asymptomatic carriers as well as in HTLV-1-associated myelopathy/Tropic spastic paraparesis (HAM/TSP) patients, suggesting that even in the absence of clinical symptoms there is a balance between the host immune surveillance and the persistent viral infection [8,9]. Therefore, to understand the regulatory mechanisms acting on HTLV-1 provirus integrated within the host genomic DNA is a key to elucidate the virological and pathophysiological aspect of HTLV-1 infection, including the mechanisms leading to transformation of the infected cells or the establishment of chronic inflammatory diseases. 

## 2. Structure of Human T-cell Leukemia Virus Type 1 (HTLV-1)

HTLV-1 is a delta-type retrovirus [10,11]. Viral RNA is reverse-transcribed, integrated into the genomic DNA of the host cell, and thereafter remains as a provirus. The size of the proviral genome is approximately 9000 base pairs (bp) [12]. As is the case with other retroviruses, there are identical sequences, long terminal repeats (LTRs), at both ends of the provirus. The 5′-LTR is the promoter for the transcripts in the sense orientation, whereas the 3′-LTR is the promoter for antisense transcription. Most of the viral structural genes, such as *gag*, *pol*, and *env* are encoded in the 5′ side of the provirus in the sense orientation, as is commonly observed in other retroviruses [13]. However, a unique characteristic of HTLV-1, also shared with the bovine leukemia virus (BLV), another delta-type retrovirus, is the presence of the pX region, which is located in the 3′ side of the provirus. There are two regulatory proteins, Tax and Rex, encoded in the pX region. Tax, the most intensively characterized viral protein, is a strong transactivator of HTLV-1 5′-LTR. Rex is another positive regulator for the expression of viral antigens, which controls the nuclear export of viral mRNAs. There are several accessory proteins also encoded in the sense orientation in the pX region, including p13, p30, p12, p27, p21Rex and p8 [14,15,16,17,18]. In addition, HTLV-1 bZIP factor (HBZ) is encoded in the pX region in the anti-sense orientation [19]. 

HTLV-1 very efficiently utilizes its small genome via alternative splicing and bidirectional transcription. The regulatory and accessory viral proteins coordinately control viral antigen expression, contributing to achieve a persistent infection with HTLV-1.

## 3. Regulation of the 5′- and 3′-LTR Promoter Regions of HTLV-1 Provirus

There is sense- and antisense-transcription from HTLV-1 provirus, driven by sequences contained in the LTRs that serve as promoters. The sequence of the 5′- and 3′-LTRs is identical, so the directionality, sense or anti-sense orientation, confers the different promoter activity on the 5′- and 3′-LTRs. There is a DNA sequence found in the promoter region of some genes (TATA-box) in the sense orientation of the LTR, but not in the anti-sense orientation. Promoters containing a TATA-box structure generally exhibit high-plasticity in their promoter activity, whereas TATA-less promoters show low transcriptional plasticity [20,21]. In line with this notion, the plus strand of the 5′-LTR, a TATA-box-containing promoter, shows variable activity, whereas the minus strand of the 3′-LTR, a TATA-less promoter, shows a relatively stable promoter activity [22]. A recent study has shown that HTLV-1 LTR possesses a bidirectional transcriptional activity and that Tax could preferentially activate the sense transcription with no or limited effect on the antisense transcription in a reporter plasmid system [23]. It is well known that the sense transcription from the 5′-LTR is significantly induced in the presence of Tax. There are three copies of imperfect repeats of a 21 bp sequence called TRE (Tax-response element) that is responsive to the transactivation mediated by the viral protein Tax. Tax is a strong positive regulator of sense transcription from the 5′-LTR [12,24,25].

A recent interesting study has further extended our understanding on how both sense and antisense transcriptions are regulated within the provirus. They showed that sense transcription from the 5′-LTR did not interfere with antisense transcription from the 3′-LTR and *vice versa* [26]. They further showed that the cell cycle arrest induced by Tax expression might inhibit Tax-mediated activation of the sense transcription without affecting antisense transcription. As the authors pointed out, the mechanism may play a role in HTLV-1 latency. The 5′-LTR is regulated by cellular signaling pathways, such as the T-cell receptor (TCR)-mediated one, in addition to the viral regulatory/accessory proteins. TCR stimulation in combination with Tax strongly enhances HTLV-1 gene expression [27]. 

On the other hand, there are several negative regulatory systems acting on the 5′-LTR during transcription, translation, and even post-translation phases. HTLV-1 p30 has the potential to inhibit the interaction between Tax and p300, resulting in suppression of the 5′-LTR [28,29]. p30 additionally enhances the retention of mRNA in the nucleus and suppresses viral antigen expression [30]. HTLV-1 p13 is also known to exert an inhibitory effect on the physical interaction between Tax and p300 [15]. Furthermore, HBZ competes with Tax for cAMP response element binding (CREB) protein and p300 binding, so HBZ suppresses the 5′-LTR [19,31,32]. The minus strand of the 3′-LTR is the promoter of the spliced form of HBZ [33,34] and is controlled by Sp-1 and Jun-D [22,35]. The unspliced form of HBZ is transcribed from the promoter located within the pX region [22].

In summary, there is convergent transcription, in the sense- and antisense-orientations, and various viral transcripts with alternative splicing within HTLV-1 provirus. Growing evidence so far indicates that HTLV-1 maintains an equilibrium between viral antigen expression and the host immune surveillance by controlling both sense and antisense transcription, viral regulatory and accessory proteins’ expression, and host cellular mechanisms, such as cellular-signaling pathways and cell cycling.

## 4. *In Vitro* and *in Vivo* Proviral Transcription Show Different Patterns

### 4.1. HTLV-1-Associated Cell Lines and Adult T-cell Leukemia (ATL)-Derived Cell Lines

It has been reported that there is clearly a distinct level of proviral expression *in vitro* and *in vivo*. Even among cell lines infected with HTLV-1, there is a wide variation in transcription level. The distinct viral gene expression should be the consequence of different cellular transformation processes occurring during *in vitro* culture or in infected individuals. In vitro T-cell immortalization by HTLV-1 is induced by a high expression level of Tax protein. As shown in previous studies, high level of tax expression is sufficient to induce immortalization of T cells [36,37,38]. Because there is no immune surveillance in *in vitro* cell culture, viral antigen expression does not confer survival disadvantage on infected cells. In contrast, adult T-cell leukemia (ATL) cell lines derived from ATL patients are generated after a long latent period *in vivo* in the presence of anti-virus immune surveillance [3,39]. In the leukemogenesis of ATL *in vivo*, the process of transformation depends not only on HTLV-1 infection but also on several other factors, such as escape from the host immune surveillance [40,41], as well as genetic and epigenetic abnormalities of the host genome [42,43,44,45,46,47,48,49,50]. As a consequence of immune pressure, ATL cell lines generally have very low or no Tax expression [39,41].

### 4.2. Fresh Peripheral Blood Mononuclear Cells (PBMC) from Infected Individuals

Transcripts with sense orientation from the 5′-LTR are generally barely detectable, if not at all, in PBMCs freshly isolated from infected individuals [41,51]. In contrast, antisense transcripts from the 3′-LTR are constitutively detectable in fresh PBMCs [34,52]. Most viral antigens are encoded in the sense transcripts from the 5′-LTR, so infected cells with high expression of viral antigens would be eliminated by the host immune surveillance. This is quite consistent with the idea that selective pressure by the host immune system is evident in naturally infected individuals. There are several mechanisms related to suppression of the 5′-LTR, including deletion of the 5′-LTR and mutations in tax gene, a strong transactivator of the 5′-LTR [40,41]. In addition, epigenetic mechanisms acting on the 5′-LTR also contribute to the silencing effect, a point which we will discuss about in detail below. Antisense transcripts are known to encode the viral antigen HBZ [19]. A possible explanation why HBZ can be expressed *in vivo* is its low immunogenicity. A previous report demonstrated that the amino acid sequence of HBZ possesses substantially low immunogenicity [53]. Another important piece of evidence about proviral expression is that *ex vivo* culture induces expression of viral proteins [54,55,56]. Although we do not know the exact mechanism for the *in vivo* silencing, it is generally accepted that HTLV-1 expression is silenced but the virus keeps its capacity to re-start production of viral particles. Recent interesting studies have shown the kinetics of the sense and antisense proviral transcription during *ex vivo* culture, suggesting the existence of a time-dependent regulatory mechanism of HTLV-1 provirus, which should be important at the initial phase of infection [57,58,59].

### 4.3. RNA-seq of Fresh ATL Cells

Recently Kataoka *et al.* published the results of a comprehensive and genome-wide sequencing analysis of fresh ATL cells from several hundreds of ATL cases in Japan [47]. The RNA-seq analysis detected not only transcription from the human genome, but also from HTLV-1 provirus integrated into the host genome of ATL cells. In line with previous reports, sense transcripts from the 5′-LTR were frequently suppressed, whereas antisense transcripts were detectable in all the ATL cases they analyzed [34,52]. Minus strand transcripts encode HBZ protein and the transcript itself has a function [19,34,60]. Accumulating evidence has suggested that HBZ plays an important role in persistent infection and pathogenesis of HTLV-1 [61,62]. Interestingly, some antisense transcripts do not terminate at the polyadenylation site of HBZ, but there are read-through transcripts into the flanking host genomic regions [34,47,63]. Although we do not know the function of such read‑through transcripts, the pattern of transcript and role of proviral transcription should be far more complicated than that of our current understanding.

## 5. Epigenetic Regulation of HTLV-1 Provirus

HTLV-1 provirus is integrated into the cellular DNA, chromatinized, and affected by genomic and epigenomic circumstances nearby the integration site [64,65,66,67]. An important characteristic of epigenetic regulation is that the epigenetic state is generally not static but variable depending on intra-cellular and/or extra-cellular conditions. For example, histone modifications are dynamically laid down and removed by chromatin-modifying enzymes [68,69]. As we discussed above, HTLV-1 has to be transcriptionally repressed but, at the same time, maintaining an ability to reactivate the proviral expression to achieve continuous infection within human beings. HTLV-1 should utilize a reversible epigenetic mechanism of the host cell to achieve a transiently suppressed condition of HTLV-1 provirus.

### 5.1. DNA Methylation of HTLV-1 Provirus

DNA methylation is a key mechanism to control gene expression of the host cells. Based on sequence analyses, HTLV-1 LTRs contain CpG islands, which are defined based on their high percentage of GC content (a GC percentage greater than 50%) and the length of the region (more than 200 bp) [70]. CpG islands are frequently present in gene promoter regions of the host cells and play a role in nucleosomal positioning and transcriptional regulation. DNA hypermethylation of CpG islands in promoter regions is associated with gene silencing. In the case of HTLV-1, DNA hypermethylation is what controls the activity of the 5′-LTR both in latently infected cell lines and ATL cells [54,71,72]. In contrast, the 3′-LTR is rarely methylated, suggesting that selective DNA methylation occurs in HTLV-1 provirus [41,71,73]. Since the sequence of the 5′- and the 3′-LTRs is identical, there should be some mechanism that induces selective DNA methylation of the 5′-LTR (or selective hypomethylation of 3′-LTR). That is a key question that remains to be answered about HTLV‑1 infection.

### 5.2. Histone Modifications in HTLV-1 Provirus

The nucleosome is the fundamental unit of chromatin, and it is composed of an octamer of the four core histones (H3, H4, H2A, and H2B). Around 147 bp of DNA wrap around each histone octamer. The histone tails are modified by acetylation, methylation, phosphorylation, and ubiquitylation [68]. These modifications function as platforms for the recruitment of specific effector proteins, such as transcriptional factors, chromatin remodelers and the general transcription apparatus, including RNA Polymerase II (RNA Pol II) [74]. Thus, histone modifications are a critical determinant for gene transcription [69]. The viral protein Tax, together with the phosphorylated CREB, recruits both CBP and p300 to the 5′-LTR [75], resulting in histone acetylation and eviction of nucleosomes, thus contributing to a strong sense transcription from the 5′-LTR [76,77]. Tax also interacts with the chromatin remodeling complex, changes nucleosome positioning, and induces transcriptional activation from the 5′-LTR [78,79]. These findings are thought to be molecular mechanisms inducing strong sense-transcription in *in vitro* cell cultures, where there is abundant Tax expression. The Tax-mediated strong transcription from the 5′-LTR is the case *in vitro* but not *in vivo*, as discussed above.

In order to characterize the pattern of histone modifications *in vivo*, we have to analyze fresh ATL cells without doing any *ex vivo* culture. There are some reports on histone acetylation and methylation of ATL cells. The previous data suggested that histone acetylation is detectable both in 5′- and 3′-LTRs [80,81]. We recently demonstrated that H3K9ac is high in the 3′-LTR but very low in the 5′-LTR in the ATL-derived cell line ED [39,67]. Another histone modification, H3K4me3, a mark of active promoter regions, also shows a similar distribution pattern as H3K9ac. In a proportion of fresh ATL cells and ATL cell lines, H3K9ac and H3K4me3 are detectable at the 5′-LTR, although the distribution is limited only to the 5′-LTR. On the other hand, the active histone marks around the 3′LTR are not limited to the LTR region, but extend to the pX region, suggesting different histone modifications between 5′- and 3′-LTRs [67].

In general, there are two categories of promoters, active and poised promoters [82]. Active promoters exhibit active histone marks both on the promoter region and downstream of the transcriptional start site, suggesting the movement of RNA Pol II starts at the promoter and moves into the gene body [74]. In contrast, poised promoters show limited distribution of H3K9ac and H3K4me3 at promoter regions. Taken together these data suggest that RNA Pol II might be present at both 5′- and 3′-LTRs. But, while the 5′-LTR is a poised promoter, the 3′-LTR functions as an active one. This is consistent with the pattern of transcription where the 3′-LTR is constitutively active but the 5′-LTR is generally suppressed. Also, poised RNA Pol II is ready to start transcription. The possibility of a poised RNA Pol II at the 5′-LTR would also explain why fresh PBMCs, isolated from HTLV-1-infected individuals, start to express Tax after a very short time in *ex vivo* culture.

Most studies on histone modifications of HTLV-1 have, so far, been limited to the promoter regions, 5′- and 3′-LTRs. However transcription is a biological event with multiple steps, including transcriptional initiation, elongation, and termination [83]. To understand the whole picture of transcriptional regulation, we need to analyze all processes of the transcription. Recent technological advances, such as chromatin immunoprecipitation sequencing (ChIP-seq) analysis will be able to provide more evidence on the epigenetic regulation of HTLV-1.

### 5.3. Insulator Region within HTLV-1 Provirus

**Figure 2:**
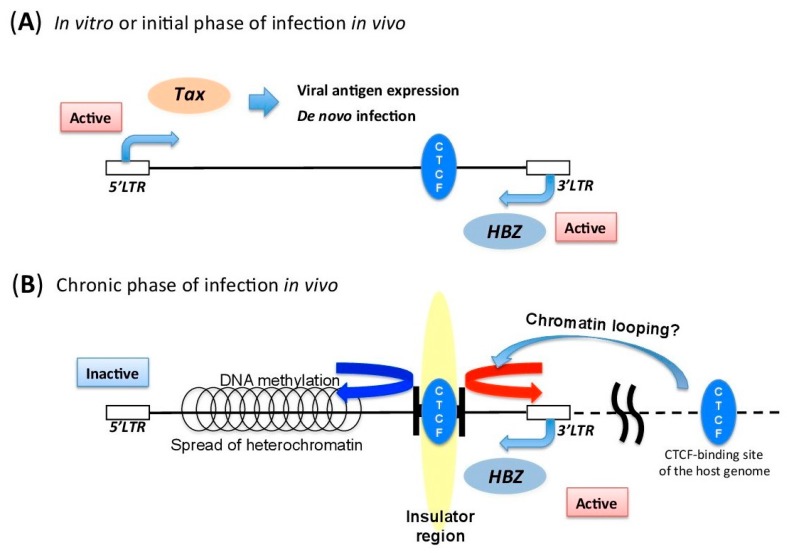
Schematic figure of mechanisms regulating HTLV-1 provirus, *in vitro* and *in vivo*. (**A**) Schematic figure of HTLV-1 provirus *in vitro* or during the initial phase of infection *in vivo.* Tax and viral structural proteins can be expressed, because there is little immune surveillance against the viral antigens. (**B**) Schematic figure of HTLV-1 provirus during the chronic phase of infection *in vivo*. HTLV-1 maintains a distinct transcription pattern between 5′ long terminal repeat (LTR) and 3′-LTR by recruiting the host insulator protein CTCF. The insulator region of HTLV-1 provirus is thought to prevent the spread of heterochromatin from 5′-LTR to 3′-LTR. This could also induce chromatin looping with the host’s CTCF-binding sites.

As mentioned above, there is a significant difference in the transcriptional activity and the epigenetic characteristics of the 5′ and the 3′ regions of HTLV-1 provirus. Since the size of the provirus is just about 9000 bp, there should be a positive regulatory mechanism to maintain such distinct transcriptional activity within the provirus [67]. We have recently reported that the insulator-binding protein CTCF (CCCTC-binding factor) directly binds to HTLV-1 provirus. Insulator regions are functional genomic regions that delimit an epigenetic border between transcriptionally active and inactive regions [84]. CTCF is also the most characterized insulator-binding protein, which is well conserved from flies to humans, and plays a fundamental role in the higher order chromatin structure of the genome [85,86]. Histone modifications, such as H3K4me3, H3K36me3, and H3K9ac, are significantly changed at the insulator region of HTLV-1. These data suggest that HTLV-1 utilizes this host insulator-binding molecule to maintain the appropriate pattern of proviral transcription to achieve persistent infection in infected individuals (Figure 2).

Another function of CTCF is chromatin loop formation by homodimerization [87]. CTCF, in concert with Cohesin, regulates the proximity of promoters and enhancers, and controls the transcriptional activity of genes [88,89,90]. This suggests the possibility that HTLV-1 provirus can form chromatin looping with distant host genomic sites through its CTCF-binding site. Therefore, CTCF‑mediated chromatin looping with the host genome is another possible mechanism to regulate proviral expression. It is very interesting that some gamma herpes viruses, such as Epstein-Barr virus (EBV) and Kaposi’s sarcoma-associated herpesvirus (KSHV), also use CTCF to switch the pattern of viral gene expression [91,92]. As is the case with HTLV-1, EBV is known as a virus with different *in vitro* and *in vivo* pattern of viral gene transcription [93]. A previous report demonstrated that CTCF plays a role in determining promoter usage at different latency states in EBV infection [92]. EBV is generally not integrated into the host genome, but HTLV‑1 is integrated as a step of the viral life cycle of retroviruses. Thus integration of HTLV-1 generates ectopic CTCF-binding sites in the human genome. This could induce aberrant chromatin structure and gene transcription of the host genome [67,94].

## 6. Integration Site and Its Role in Proviral Transcription

In principle, each infected clone has a different integration site (IS). IS data has been used to identify clonality of HTLV-1-infected cells. According to technological advances in DNA detection and sequencing, current analysis of clonality of infected cells is becoming far more accurate and quantitative than before [64,95,96,97,98,99]. Application of modified PCR and DNA sequencing enables us to determine the exact position of the viral integration site within the host genome. Novel DNA sequencing technologies have also enabled the characterization of the nature of HTLV-1 integration landscape with high resolution [66]. Now, we can characterize the distribution of integration sites and quantify the degree of clonal expansion of each individual infected cell with extremely high resolution by using next-generation sequencing technology. We know there are approximately ten of thousands of infected clones in a typical infected individual [66].

The distribution of HTLV-1 integration sites in the infected individuals is determined by two key factors, *i.e.*, initial integration-targeting and preferential survival of infected clones *in vivo*. There is clear difference in HTLV-1 IS between *in vitro*-infected samples and *in vivo*-infected clinical samples [64]. This idea is consistent with a recent report on the clonality analysis of cells infected with BLV, another member of the delta type retroviruses. The study showed significant depletion of BLV proviral clones located in transcriptionally active genomic sites during primary infection [100]. The data suggests that transcriptionally active regions are preferentially targeted by BLV integration, but the provirus integrated in transcriptionally active regions tends to express a viral antigen, which should result in removal by the host immune surveillance. During persistent infection *in vivo*, the infected clone that has an advantage in escaping from the host immune surveillance would be able to live for a long time. Although there is no clear hot spot of IS associated with development of ATL, there are some tendencies as listed below [64,65].

(i) HTLV-1 tends to be integrated into open chromatin.
(ii) Infected clones with ISs within a gene and with the same orientation as the host gene tend to expand.
(iii) Presence of a transcription factor- or histone modifier-binding site, such as Brg1 and STAT-1, near the IS can affect the frequency of spontaneous Tax expression in *ex vivo* culture.

The data has also demonstrated that spontaneous Tax expression in *ex vivo* culture is inversely correlated with clonal abundance of infected clones, suggesting that Tax expression is not necessarily related with clonal expansion of infected cells *in vivo* [65]. Taken together, all these findings have demonstrated that the IS plays a role in proviral gene transcription.

## 7. Deletions and Mutations in HTLV-1 Proviral Genome

Compared with HIV-1 provirus, the HTLV-1 genome is highly conserved, which is evidence for the idea that HTLV-1 maintains its viral copy number via clonal expansion of infected cells rather than *de novo* infection with viral particles, in which error-prone reverse transcriptase generates genetic mutations in the viral genome [101]. Even so, evidence on deletions of HTLV-1 proviral genome has been provided [102,103,104,105] by several reports on defective HTLV-1 proviruses. There are two types, type 1 and type 2, of defective proviruses. Type 1 defective proviruses lack genomic segments within the provirus, whereas the type 2 lack the 5′-LTR [104]. Both types of defective proviruses should have less capacity to produce viral antigens than the complete ones, so the presence of the defective provirus is thought to be a consequence of selection by the host immune surveillance. Genetic mutations of the viral genome encoding viral genes were also reported previously. Furukawa *et al.* showed the presence of point mutations with a premature stop codon in *tax* gene in ATL patients [40]. Fan *et al.* performed a comprehensive analysis and found that there are mutations in various viral genes but not in the *HBZ* gene [106]. Such deletions and mutations of the provirus should significantly affect expression of viral genes.

## 8. Closing Remarks

Recent HTLV-1 research has made substantial progress in proviral regulation, but there are several issues that remain to be addressed. For example, the evidence we have so far is the result of analyzing a population of infected cells or ATL cells, so we cannot exclude the possibility that a part of the infected cells sporadically expresses plus-strand transcripts like Tax at the single-cell level. Also, it is still unclear how much the maintenance of ATL cells depends on HTLV-1 provirus. It is obvious that genetic and epigenetic alterations associated with oncogenesis in the host genome play a central role in the leukemogenesis of ATL [42,43,44,45,46,47,48,49,50]. Given that the incidence of T-cell malignancy in peripheral CD4 T cell is quite low in the absence of HTLV-1 infection, HTLV-1 does play a role in ATL generation in HTLV-1 infection. Intermittent expression of Tax possibly accelerates genomic and epigenomic abnormalities in the host cellular genome [25,107]. Continuous antisense transcription from the 3′-LTR supports the proliferation of ATL cells [34,52], which gives the host cells more of a chance to accumulate genomic and epigenomic alterations [3,13,61]. A wide variability of leukemogenesis among individual ATL cases is likely to exist. Further investigations are required to elucidate the role of proviral transcription in HTLV-1 pathogenesis.
